# Identification of Imidazole–Benzenesulfonamide Derivatives with Anti-Inflammatory Activity Through Modulation of NF-κB Signaling

**DOI:** 10.3390/molecules31193472

**Published:** 2026-09-29

**Authors:** Povilas Kavaliauskas, Valdas Vainauskas, Vidmantas Petraitis, Ruta Petraitiene, Waldo Acevedo, Ramunė Grigalevičiūtė, Birutė Grybaitė, Božena Golcienė, Rolandas Stankevičius, Julius Rakickas, Vytautas Mickevičius

**Affiliations:** 1 Institute for Genome Sciences, School of Medicine, University of Maryland, 655 W. Baltimore Street, Baltimore, MD 21201, USA; povilas.kavaliauskas@som.umaryland.edu; 2 Department of Organic Chemistry, Kaunas University of Technology, Radvilėnų Rd. 19, LT-50254 Kaunas, Lithuania; 3 Institute of Infectious Diseases and Pathogenic Microbiology, Birštono Str. 38A, LT-59116 Prienai, Lithuania; 4 Biological Research Center, Lithuanian University of Health Sciences, Tilzes Str. 18/7, LT-47181 Kaunas, Lithuania; 5 Center for Discovery and Innovation, Hackensack Meridian Health, Nutley, NJ 07110, USA; 6 Institute of Chemistry, Faculty of Sciences, Pontificia Universidad Católica de Valparaíso, Valparaíso 2373223, Chile; waldo.acevedo@pucv.cl; 7 Center for Interdisciplinary Research in Biomedicine, Biotechnology and Well-Being (CID3B), Pontificia Universidad Católica de Valparaíso, Valparaíso 2340025, Chile; 8 Department of Animal Nutrition, Lithuanian University of Health Sciences, Tilzes Str. 18, LT-47181 Kaunas, Lithuania; 9 Public Institution Šilutė Primary Health Care Center, Rusnes Str. 1, LT-99185 Šilutė, Lithuania

**Keywords:** anti-inflammatory activity, NF-κB, imidazole–benzenesulfonamide derivatives

## Abstract

Inflammatory disorders remain an important therapeutic challenge, highlighting the need to identify novel small molecules capable of modulating inflammatory signaling. In this study, an in-house library of aromatic and heterocyclic drug-like compounds was screened for inhibition of lipopolysaccharide (LPS)-induced nuclear factor kappa B (NF-κB) activation using THP-1 dual-reporter cells. Eight compounds markedly suppressed NF-κB activity, and five structurally related 4-phenylimidazole-based benzenesulfonamide derivatives (**1232**, **1239**, **1241**, **1242**, and **1245**) were prioritized based on their cytotoxicity profiles. Functional activity was subsequently evaluated in LPS-stimulated RAW 264.7 macrophages. The selected compounds reduced nitrite production, attenuated LPS-induced nuclear localization of NF-κB, and differentially affected IL-6, KC/CXCL1, and TNF-α production. The naphthyl-substituted derivative **1239** showed prominent inhibition of nitrite, IL-6, and KC/CXCL1 production, whereas the ethylthio derivative **1232** preferentially reduced TNF-α. Molecular docking against a panel of inflammation-associated proteins revealed favorable predicted interactions of compound **1239** with IκB kinase(IKK) and p38 mitogen-activated protein kinase (p38 MAPK), with docking scores of −10.3 kcal/mol for both proteins and binding poses overlapping the corresponding crystallographic ligand-binding regions. Collectively, these findings identify 4-phenylimidazole-based benzenesulfonamides as a promising chemical series with anti-inflammatory activity and suggest IKK and p38 MAPK as potential components of the affected signaling network. Further structure–activity and mechanistic studies are required to define the molecular targets and optimize the activity of this compound class.

## 1. Introduction

Inflammation is an essential component of the host response to infection and tissue injury and is required for the elimination of harmful stimuli and restoration of tissue homeostasis [1]. When excessive or inadequately resolved, inflammatory responses can promote tissue damage and contribute to the development and progression of chronic inflammatory, autoimmune, metabolic, and malignant diseases [2,3]. Consequently, identification of compounds capable of controlling excessive inflammatory signaling remains an important area of drug discovery [4].

Nuclear factor kappa B (NF-κB) is one of the principal transcriptional regulators of the inflammatory response. In resting cells, NF-κB proteins are retained predominantly in the cytoplasm through association with inhibitory IκB proteins [5]. Activation of the canonical pathway promotes IκB phosphorylation and degradation, allowing NF-κB, particularly the p50/RelA (p65) complex, to translocate to the nucleus and regulate transcription of genes involved in inflammation and immune responses [4]. These include genes encoding inflammatory cytokines, chemokines, adhesion molecules, and enzymes involved in the generation of inflammatory mediators [6]. Dysregulated NF-κB activity is therefore closely associated with the persistence of inflammatory responses and represents an established target for pharmacological intervention.

Lipopolysaccharide (LPS), a major component of the outer membrane of Gram-negative bacteria, provides a well-established experimental stimulus for investigating inflammatory signaling. Recognition of LPS by the Toll-like receptor 4 (TLR4) complex initiates intracellular signaling that activates NF-κB and other transcriptional pathways [7]. In macrophages, this response results in the production of inflammatory cytokines and induction of enzymes such as inducible nitric oxide synthase (iNOS), leading to increased nitric oxide production. LPS-stimulated macrophages are consequently widely used for the functional evaluation of candidate anti-inflammatory molecules [7,8].

Despite extensive investigation of NF-κB signaling, development of therapeutically useful pathway inhibitors remains challenging. NF-κB has important physiological functions in immunity, cell survival, tissue repair, and inflammatory resolution, and broad inhibition of the pathway may therefore produce undesirable effects [9]. This provides a rationale for continued screening of chemically diverse small molecules capable of modulating NF-κB-dependent inflammatory responses and for identifying structural classes that may provide starting points for further optimization.

Heterocyclic compounds represent an important source of pharmacologically active molecules, and imidazole-containing derivatives in particular have shown diverse biological activities [10,11]. In the context of inflammation, substituted pyridinyl-imidazoles have been extensively investigated as ATP-competitive inhibitors of p38 MAPK, a kinase involved in the regulation of pro-inflammatory cytokine production, and several p38-directed inhibitors have progressed into clinical development [12,13]. For example, the pyridinyl-imidazole p38 inhibitor RWJ-67657 was evaluated in healthy volunteers and attenuated endotoxin-induced TNF-α, IL-6, and IL-8 responses, providing clinical proof-of-concept for this chemotype [14]. Clinically used azole drugs containing related heterocyclic motifs, including several antifungal agents, have also been reported to exhibit ancillary anti-inflammatory effects, although inflammation is not their primary therapeutic indication [15]. On a pre-clinical level, imidazol-5-yl pyridine derivatives targeting p38α/MAPK14 reduced inflammatory mediator production in RAW 264.7 macrophages, while imidazole-containing emodin derivatives suppressed iNOS, cytokine production, and NF-κB signaling [16,17].

The in-house library used in this study comprised structurally diverse aromatic and heterocyclic compounds, including imidazole- and sulfonamide-containing derivatives with previously demonstrated biological activity. Several members of this library had previously been evaluated for antiproliferative activity in cancer cell models. Because NF-κB signaling contributes not only to inflammatory responses but also to cellular survival and proliferation, we hypothesized that compounds with established biological activity in cancer models might also modulate NF-κB-dependent inflammatory signaling. Accordingly, the library was subjected to an unbiased phenotypic screen for inhibition of LPS-induced NF-κB activation.

In the present study, an in-house library comprising 85 structurally diverse aromatic and heterocyclic drug-like small molecules was screened for compounds capable of suppressing LPS-induced NF-κB activation. Primary hits were subjected to cytotoxicity counter-screening, followed by functional characterization of selected 4-phenylimidazole-based benzenesulfonamide derivatives in LPS-stimulated macrophages. Their effects on nitric oxide production, NF-κB nuclear localization, and inflammatory cytokine secretion were subsequently examined to establish whether the activity detected in the reporter screen translated into modulation of macrophage inflammatory responses.

## 2. Results

### 2.1. Identification of Compounds Suppressing LPS-Induced NF-κB Activation

**Figure 1:**
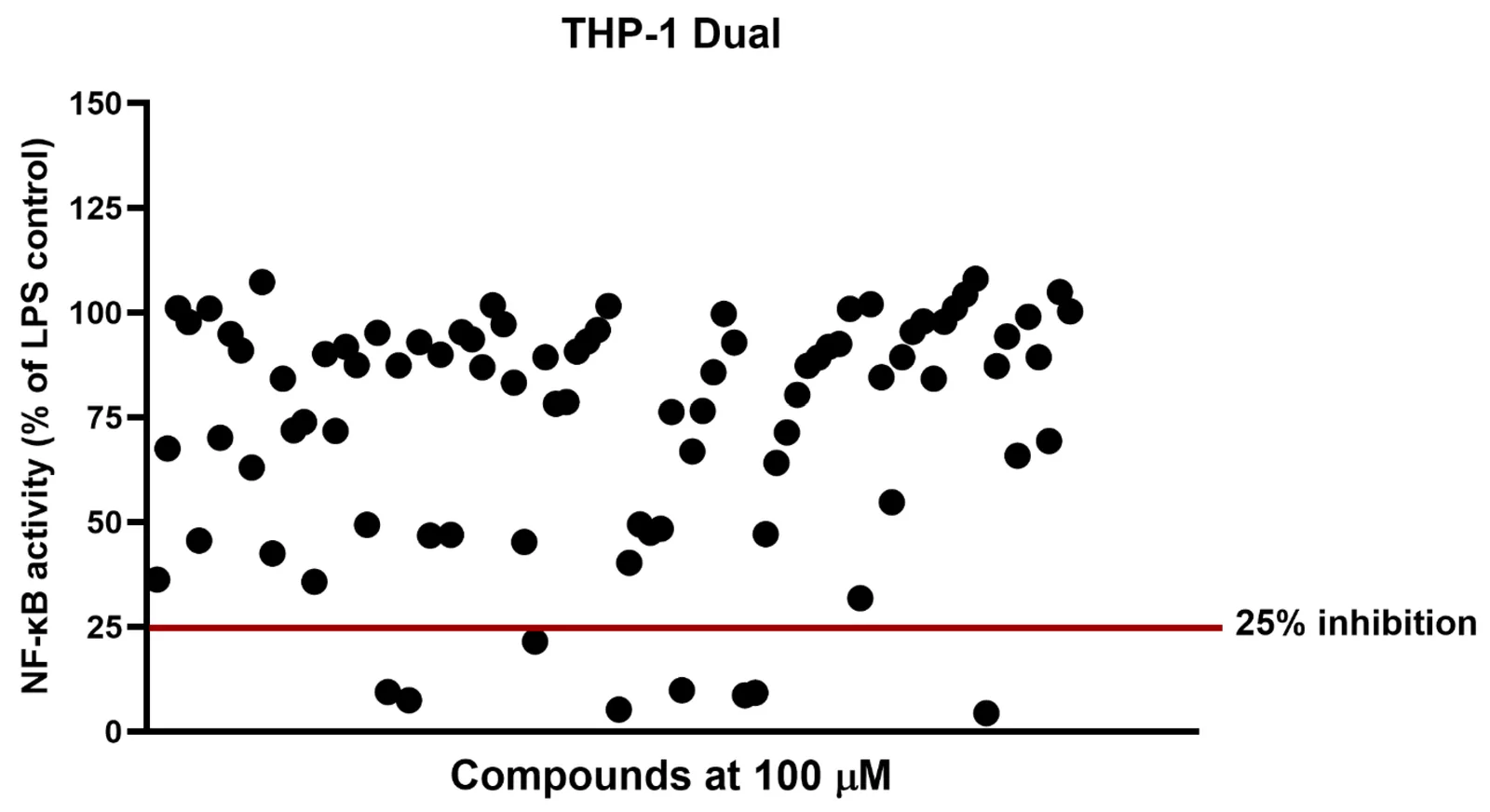
Screening of the small-molecule library consisting of 85 structurally diverse compounds for inhibition of LPS-induced NF-κB activity. THP-1 Dual cells were treated with compounds at 100 μM and stimulated with LPS. NF-κB reporter activity was normalized to the LPS-stimulated control. The horizontal line denotes the cutoff used for primary hit selection (25% residual NF-κB activity). Each dot represents the mean % inhibition calculated from three independent experimental replicates.

An in-house library consisting of 85 drug-like small molecules, comprising structurally diverse aromatic and heterocyclic compounds, was screened for anti-inflammatory activity using THP-1 dual-reporter cells. Cells were exposed to individual compounds at 100 μM, stimulated with LPS, and NF-κB reporter activity was measured relative to the LPS-stimulated control. Most compounds had little or moderate effect on the reporter response, whereas a smaller group markedly reduced LPS-induced NF-κB activation (Figure 1). Compounds resulting in residual NF-κB activity below 25% of the LPS control were considered primary hits and selected for further analysis.

**Figure 2:**
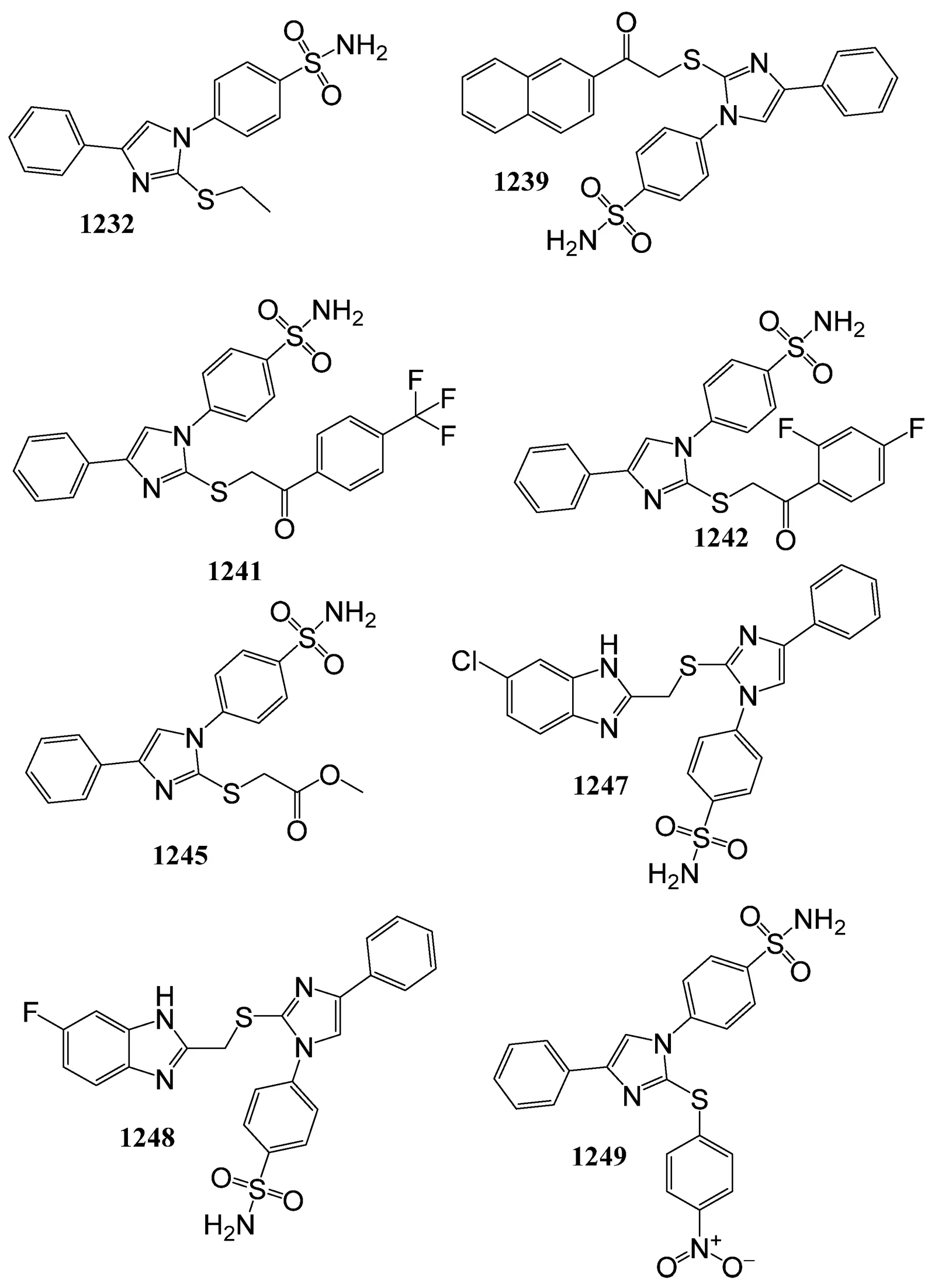
Chemical structures of the eight compounds selected from the primary NF-κB reporter screen: **1232**, **1239**, **1241**, **1242**, **1245**, **1247**, **1248**, and **1249**.

The eight primary hits, **1232**, **1239**, **1241**, **1242**, **1245**, **1247**, **1248**, and **1249**, are shown in Figure 2. The synthesis and antiproliferative characterization of these compounds were previously reported [18] as part of a series of sulfonamide–imidazole hybrid derivatives (Table S1). Compounds **1232**, **1239**, **1241**, **1242**, and **1245** share a 4-phenylimidazole core bearing a *p*-sulfamoylphenyl substituent and a sulfur-linked group at the imidazole 2-position.

The sulfur-linked substituent at the 2-position of the imidazole ring is represented by an ethyl group in **1232**, aryl ketone-containing substituents in **1239**, **1241**, and **1242**, and a methyl acetate moiety in **1245**. Compounds **1247** and **1248** contain a benzimidazole-based scaffold linked through sulfur to a substituted imidazole ring, while **1249** is an imidazole derivative containing sulfonyl-substituted aromatic groups. Thus, despite structural variation, the primary hits are characterized by nitrogen-containing aromatic heterocycles together with sulfur-containing substituents.

**Figure 3:**
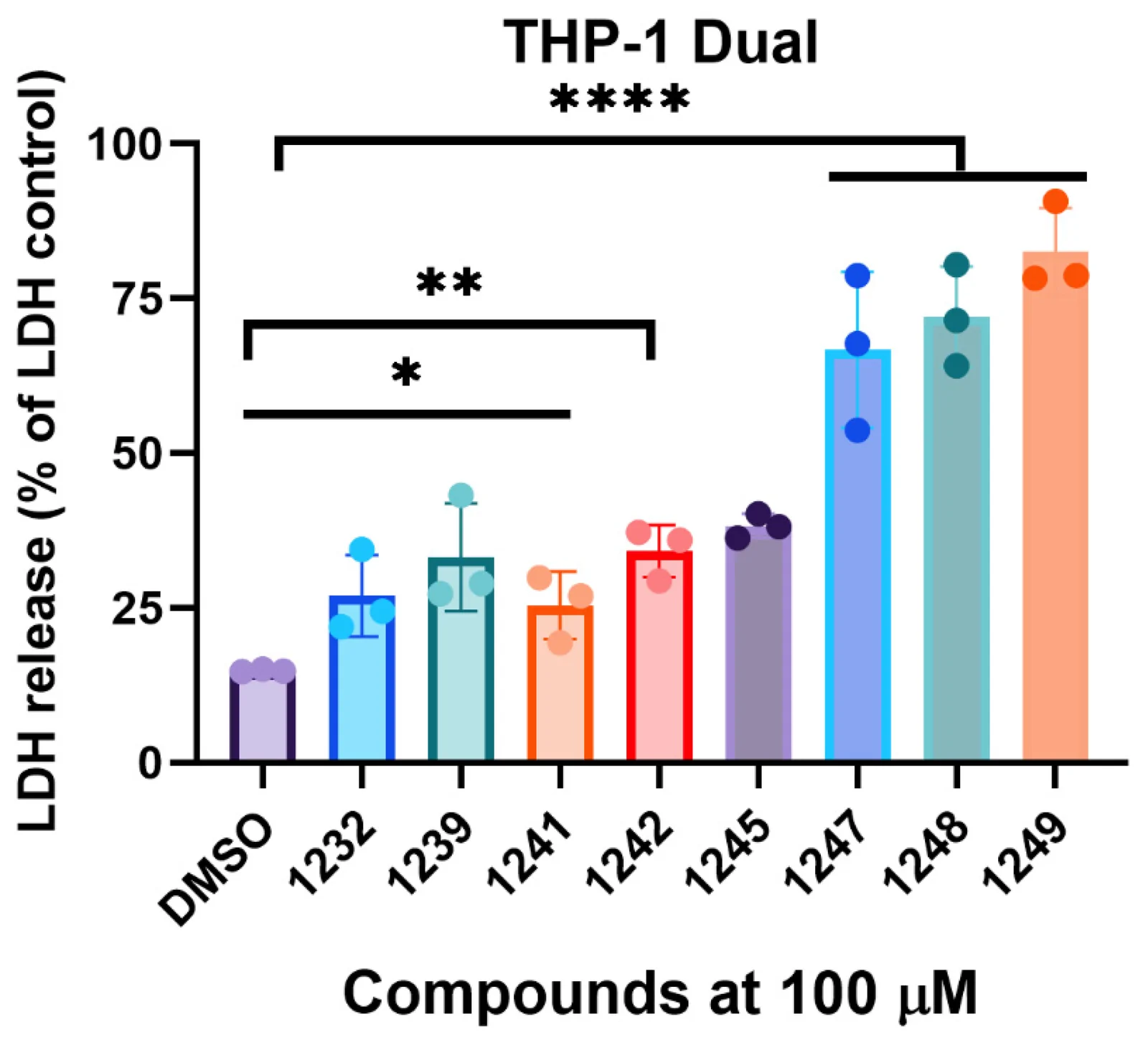
Cytotoxicity of compounds identified in the primary NF-κB screen. THP-1 Dual cells were treated with the indicated compounds at 100 μM, and LDH release to tissue culture media was measured as an indicator of cytotoxicity. Data are expressed relative to the LDH positive control and shown as mean ± SD of three replicates. * *p* < 0.05, ** *p* < 0.01, **** *p* < 0.0001.

To determine whether reduced NF-κB activity was associated with cytotoxicity, the eight hits were evaluated by LDH release at 100 μM (Figure 3). Compounds **1247**, **1248**, and **1249** caused substantial LDH release and were excluded from further analysis. In contrast, **1232**, **1239**, **1241**, **1242**, and **1245** showed considerably lower cytotoxicity under the same conditions. Based on their NF-κB inhibitory activity and LDH profiles, these five compounds were selected for subsequent characterization.

To further evaluate the concentration dependence of NF-κB inhibition, compounds **1239**, **1241**, and **1245** were tested across a range of concentrations in THP-1 dual-reporter cells (Figure S1). These compounds were prioritized based on their strong activity in the initial screen and comparatively low cytotoxicity in the LDH release assay. All three compounds produced concentration-dependent inhibition of NF-κB reporter activity (Figure S1). Compound **1239** showed the greatest activity, with an IC_50_ of 0.680 µM, followed by compound **1241** with an IC_50_ of 0.933 µM, whereas compound **1245** was less potent, with an IC_50_ of 1.746 µM.

Collectively, these data identified a subset of compounds that strongly suppressed LPS-induced NF-κB activation while exhibiting comparatively limited cytotoxicity. Based on these findings, compounds **1232**, **1239**, **1241**, **1242**, and **1245** were selected for further evaluation of their anti-inflammatory activity, while compounds **1239**, **1241**, and **1245** demonstrated low micromolar IC_50_ values.

### 2.2. Selected Compounds Suppress LPS-Induced Nitric Oxide Production in RAW 264.7 Macrophages

To determine whether the inhibitory activity observed in the THP-1 dual-reporter system translated into a functional anti-inflammatory response, the five selected compounds were further evaluated in RAW 264.7 macrophages [19]. Cells were treated with compounds **1232**, **1239**, **1241**, **1242**, and **1245** at 100 μM and stimulated with LPS overnight. Nitric oxide (NO) production was assessed indirectly by measuring nitrite accumulation in the culture supernatant using the Griess assay.

**Figure 4:**
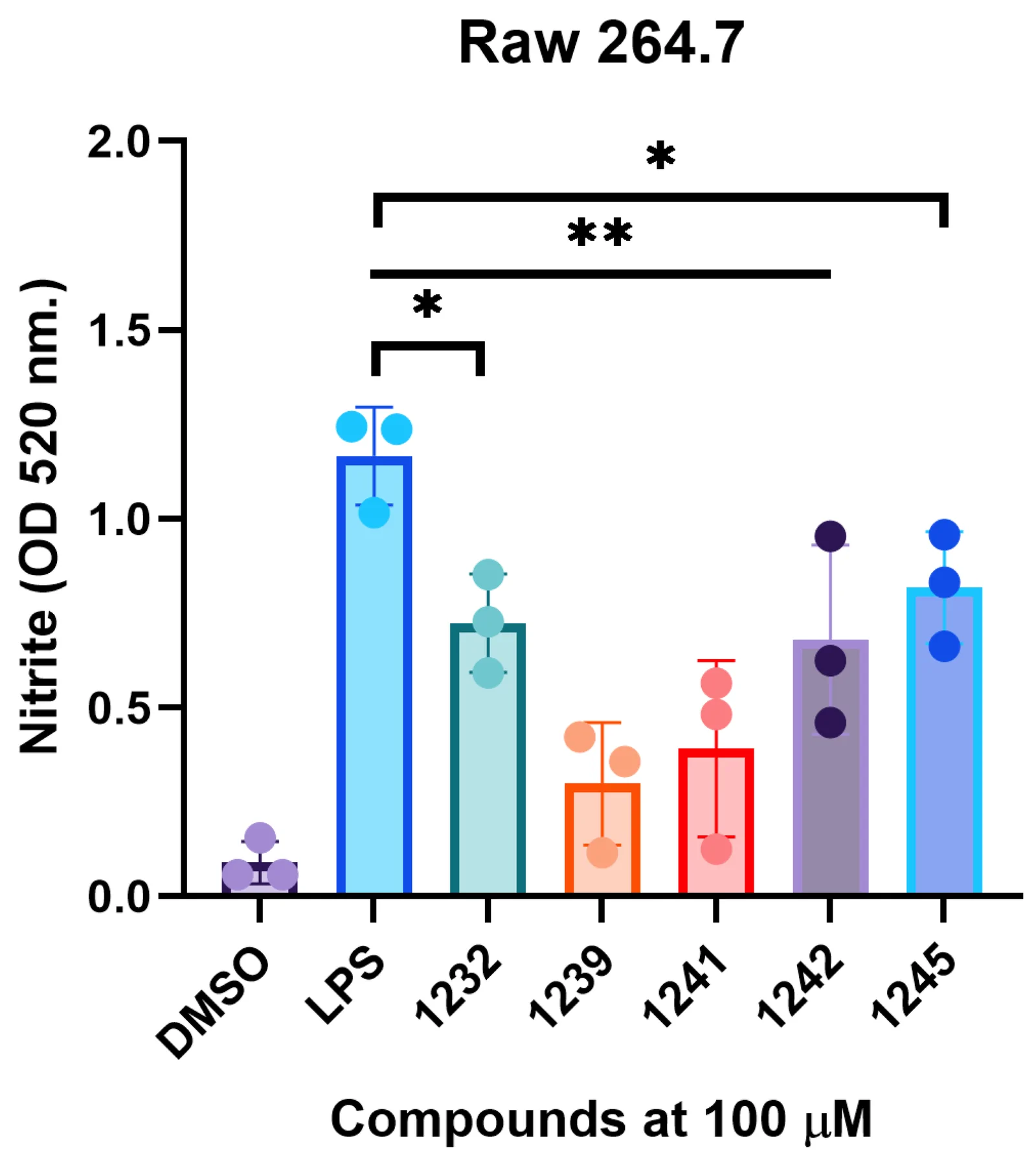
Effects of selected compounds on LPS-induced nitrite production and RAW 264.7 macrophage morphology. RAW 264.7 macrophages were treated with the indicated compounds at 100 μM and stimulated with LPS overnight. Nitrite accumulation in culture supernatants was determined using the Griess assay and expressed as absorbance at 520 nm. Data are presented as mean ± SD of three replicates. * *p*< 0.05, ** *p* < 0.01.

LPS stimulation resulted in a marked increase in nitrite production compared with the unstimulated control (Figure 4). All five compounds reduced nitrite accumulation to varying degrees. The strongest reduction was observed with compounds **1239** and **1241**, while compounds **1232**, **1242**, and **1245** produced moderate effects. These results indicate that the activity identified in the NF-κB reporter screen is accompanied by reduced production of a downstream inflammatory mediator in LPS-activated macrophages.

Cell morphology was examined in parallel by phase-contrast microscopy (Figure 4). Macrophages treated with compounds **1232**, **1239**, and **1241** retained morphology comparable to that of vehicle-treated cells. More pronounced morphological changes were observed following treatment with compounds **1242** and **1245**, although extensive cell detachment or loss of the monolayer was not apparent.

Collectively, these findings demonstrate that the selected 4-phenylimidazole-based benzenesulfonamide derivatives suppress LPS-induced inflammatory responses beyond the THP-1 reporter system. Among these derivatives, compounds **1239** and **1241**, bearing naphthyl- and trifluoromethylphenyl-substituted thioacetyl moieties, respectively, produced the strongest reduction in nitrite accumulation in RAW 264.7 macrophages.

### 2.3. Selected Compounds Alter NF-κB Nuclear Localization and LPS-Induced Cytokine Production

**Figure 5:**
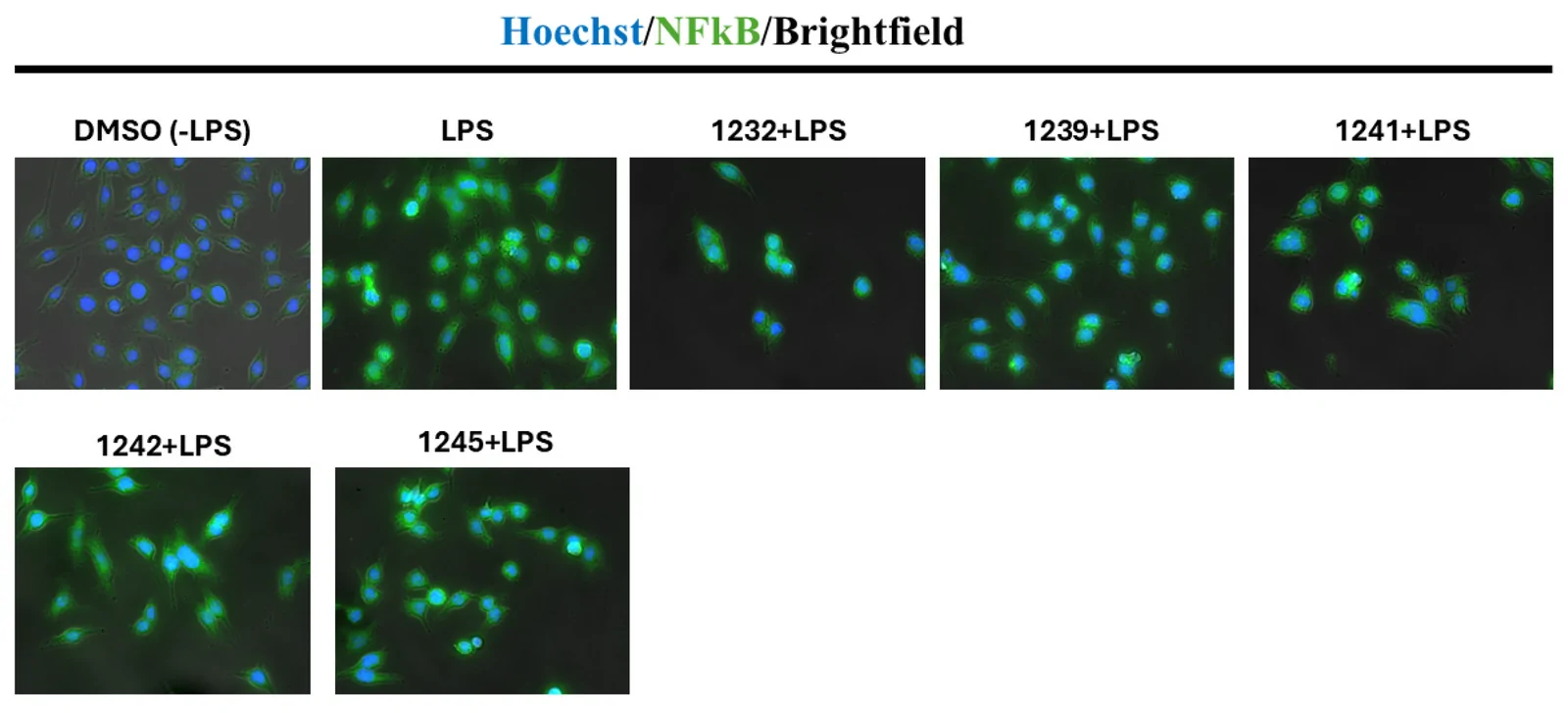
Effect of selected compounds on LPS-induced NF-κB localization in RAW 264.7 macrophages. Cells were pretreated with the indicated compounds at 100 μM for 1 h and subsequently stimulated with LPS for 3 h. NF-κB localization was examined by immunofluorescence microscopy. Nuclei are stained with Hoechst (blue), NF-κB is shown in green, and brightfield images are included in the composite.

To further examine the effects of the selected compounds on NF-κB signaling, NF-κB localization was evaluated in RAW 264.7 macrophages by immunofluorescence microscopy. Cells were pretreated with compounds **1232**, **1239**, **1241**, **1242**, and **1245** at 100 μM for 1 h, followed by LPS stimulation for 3 h. In unstimulated cells, NF-κB staining was predominantly extranuclear, whereas LPS stimulation resulted in pronounced accumulation of NF-κB within Hoechst-positive nuclei (Figure 5).

Pretreatment with the compounds altered this localization pattern and reduced the apparent nuclear accumulation of NF-κB. The effect was particularly evident for compound **1239** and compound **1241**. These observations were consistent with the inhibitory activity detected in the THP-1 dual-reporter assay and the reduction in nitrite production observed in LPS-stimulated macrophages.

**Figure 6:**
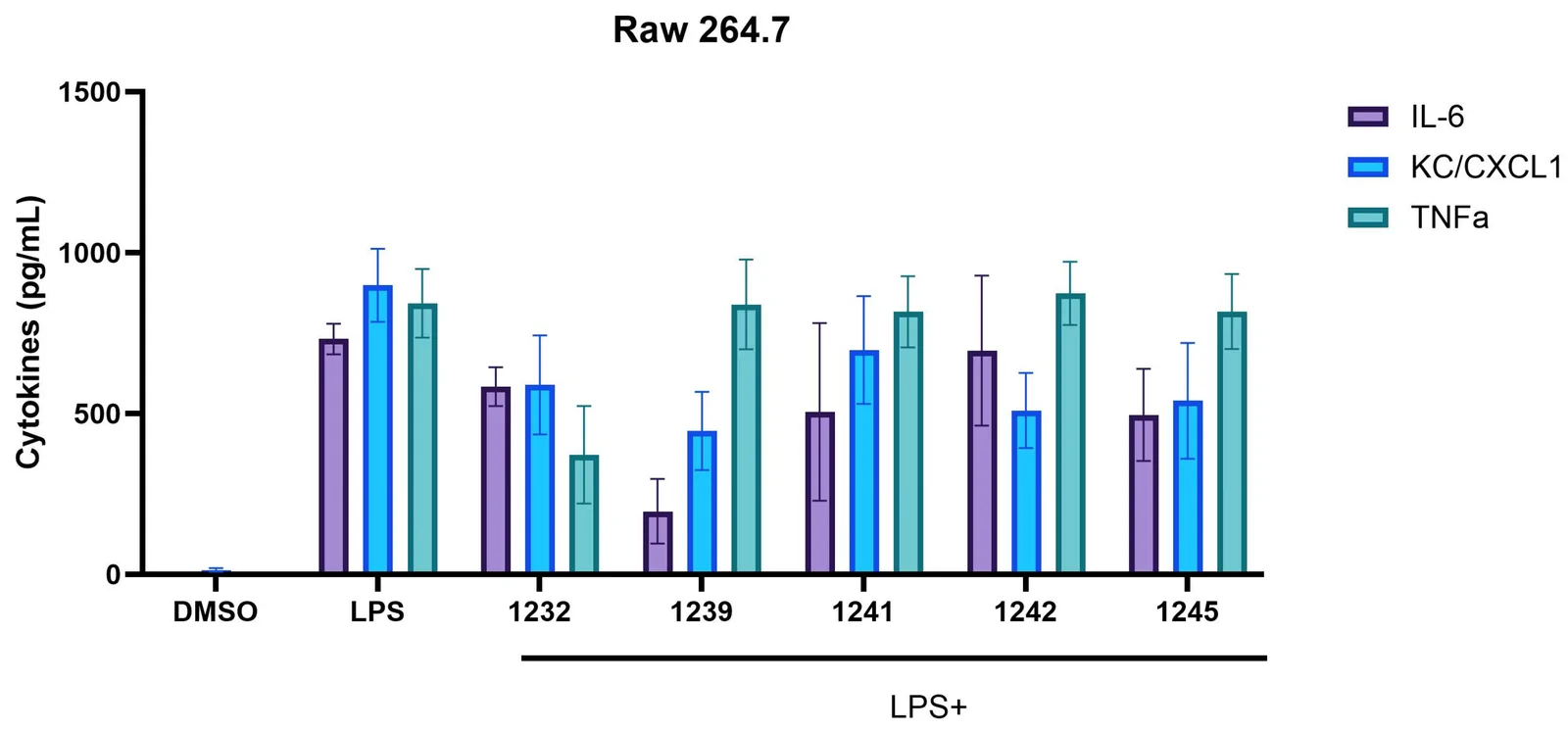
Effect of selected compounds on LPS-induced cytokine production in RAW 264.7 macrophages. Cells were pretreated with the indicated compounds at 100 μM for 1 h and subsequently stimulated with LPS for 24 h. IL-6, KC/CXCL1, and TNF-α concentrations in culture supernatants were determined by ELISA. Data are presented as mean ± SD (*n* = 3 technical replicates).

We next determined whether these effects were accompanied by changes in inflammatory cytokine production. RAW 264.7 macrophages were pretreated with the compounds for 1 h, followed by LPS stimulation for 24 h, and IL-6, KC/CXCL1KC/CXCL1 and TNF-α concentrations were measured in culture supernatants (Figure 6). LPS stimulation resulted in mean IL-6, KC/CXCL1KC/CXCL1, and TNF-α concentrations of 731.9, 898.9, and 842.9 pg/mL, respectively.

The most pronounced effect on IL-6 was observed with compound **1239**, which reduced IL-6 from 731.9 to 196.8 pg/mL (73.1% reduction; *p* = 0.008). Compound **1232** reduced IL-6 to 584.1 pg/mL, while compounds **1241**, **1242**, and **1245** resulted in concentrations of 505.6, 696.3, and 496.0 pg/mL, respectively; these differences did not reach statistical significance following multiple-comparison correction.

A broader inhibitory effect was observed for KC/CXCL1KC/CXCL1. Compound **1239** reduced KC/CXCL1KC/CXCL1 from 898.9 to 446.4 pg/mL (50.3%; *p* = 0.010). Significant reductions were also observed with derivative **1242** (509.7 pg/mL; 43.3% reduction; *p* = 0.025) and **1245** (539.9 pg/mL; 39.9% reduction; *p* = 0.039). Compounds **1232** and **1241** reduced mean KC/CXCL1KC/CXCL1 concentrations to 589.2 and 698.0 pg/mL, respectively, although these differences were not statistically significant.

A distinct activity profile was observed for TNF-α. Compound **1232** reduced TNF-α production from 842.9 to 372.3 pg/mL, corresponding to a 55.8% reduction (*p* = 0.002). In contrast, compounds **1239**, **1241**, **1242**, and **1245** had little effect on TNF-α production, with mean concentrations remaining between 816.5 and 874.1 pg/mL.

**Figure 7:**
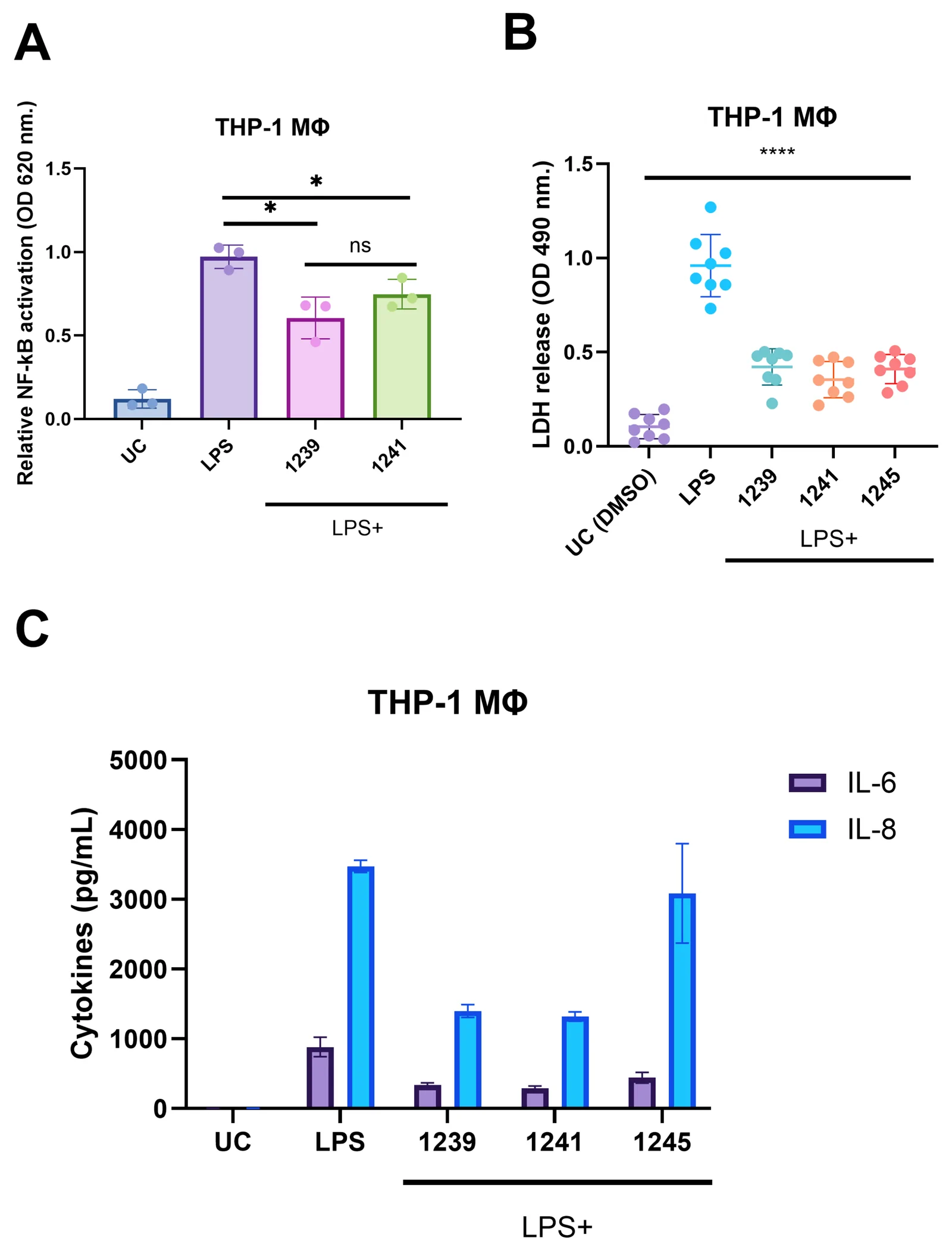
Anti-inflammatory activity and cytotoxicity of selected compounds in differentiated THP-1 macrophages. (**A**) Effect of compounds **1239** and **1241** on LPS-induced NF-κB activation in THP-1 macrophages. NF-κB reporter activity was measured at 620 nm. (**B**) Cytotoxicity of compounds **1239**, **1241**, and **1245** assessed by LDH release at 490 nm. (**C**) Effect of compounds **1239**, **1241**, and **1245** on LPS-induced IL-6 and IL-8 production. Cytokine concentrations were measured in culture supernatants and expressed as pg/mL. Data are presented as mean ± SD. Statistical significance is indicated as * *p* < 0.05 and **** *p* < 0.0001; ns, not significant. UC, untreated control; LPS, lipopolysaccharide.

Building on the cytokine phenotype observed in murine macrophages, we next sought to determine whether the anti-inflammatory activity of the prioritized compounds was reproduced in a human macrophage model. Differentiated THP-1 macrophages were stimulated with LPS in the presence of selected compounds, and NF-κB activity, LDH release, and cytokine production were assessed (Figure 7). LPS stimulation markedly increased NF-κB reporter activity compared with the unstimulated control. Treatment with compound **1239** significantly reduced LPS-induced NF-κB activation, while compound **1241** produced a more moderate reduction (Figure 7A). Importantly, treatment with compounds **1239**, **1241**, and **1245** did not increase LDH release relative to the LPS-stimulated control; instead, LDH levels were lower in compound-treated cultures, indicating that the observed suppression of inflammatory responses was not accompanied by increased cytotoxicity under these conditions (Figure 7B).

Consistent with the cytokine-modulatory phenotype observed in murine macrophages, LPS induced robust production of IL-6 and IL-8 in differentiated THP-1 macrophages (Figure 7C). Compounds **1239** and **1241** substantially reduced both cytokines, with particularly pronounced decreases in IL-8 production. In contrast, compound **1245** produced a more modest reduction in IL-6 and had comparatively little effect on IL-8. These findings extend the activity of the prioritized derivatives to a human macrophage model and demonstrate that compounds **1239** and **1241** retain a broader anti-inflammatory phenotype across murine and human cellular systems.

Collectively, these data show that the 4-phenylimidazole-based benzenesulfonamide derivatives do not uniformly suppress LPS-induced inflammatory responses but instead display compound- and mediator-dependent activity across murine and human macrophage models. In RAW 264.7 macrophages, the naphthyl-substituted derivative **1239** showed prominent activity against IL-6 and KC/CXCL1, whereas the ethylthio derivative **1232** preferentially reduced TNF-α. Consistent with this broader activity profile, compounds **1239** and **1241** also reduced LPS-induced NF-κB activation and attenuated IL-6 and IL-8 production in differentiated THP-1 macrophages, while **1245** showed a more limited effect. Together, these findings indicate that substitution at the sulfur-linked position influences the inflammatory response profile of this compound series and that the activity of selected derivatives is retained across both murine and human macrophage systems.

### 2.4. Molecular Docking Suggests IKK and p38 MAPK as Potential Interaction Sites of the Selected Compounds

To explore and propose potential molecular interactions that could contribute to the observed anti-inflammatory phenotype, molecular docking was performed against a panel of proteins selected to represent key nodes in LPS-associated inflammatory signaling, including proximal TLR4 pathway components, regulators of NF-κB activation, MAPK pathway kinases, and other inflammation-associated proteins [20,21]. Compounds **1232**, **1239**, **1241**, **1242**, and **1245** were evaluated against COX-2, TLR4, MyD88, IKK, p38 MAPK, NF-κB, ERK2, MEK1, TRKA, and TPK [22,23,24,25,26]. The compounds displayed variable predicted affinities across the target panel, with several derivatives showing favorable docking scores toward kinases associated with NF-κB and MAPK signaling (Table 1).

Among the compounds examined, **1239** showed predicted binding energies of −10.3 kcal/mol for both IKK and p38 MAPK. Compound **1241** also showed favorable predicted interactions with IKK and p38 MAPK, with docking scores of −9.6 and −9.7 kcal/mol, respectively. Compound **1242** yielded a predicted binding energy of −9.6 kcal/mol for IKK and −9.1 kcal/mol for p38 MAPK, whereas weaker interactions with these proteins were predicted for compounds **1232** and **1245**.

**Figure 8:**
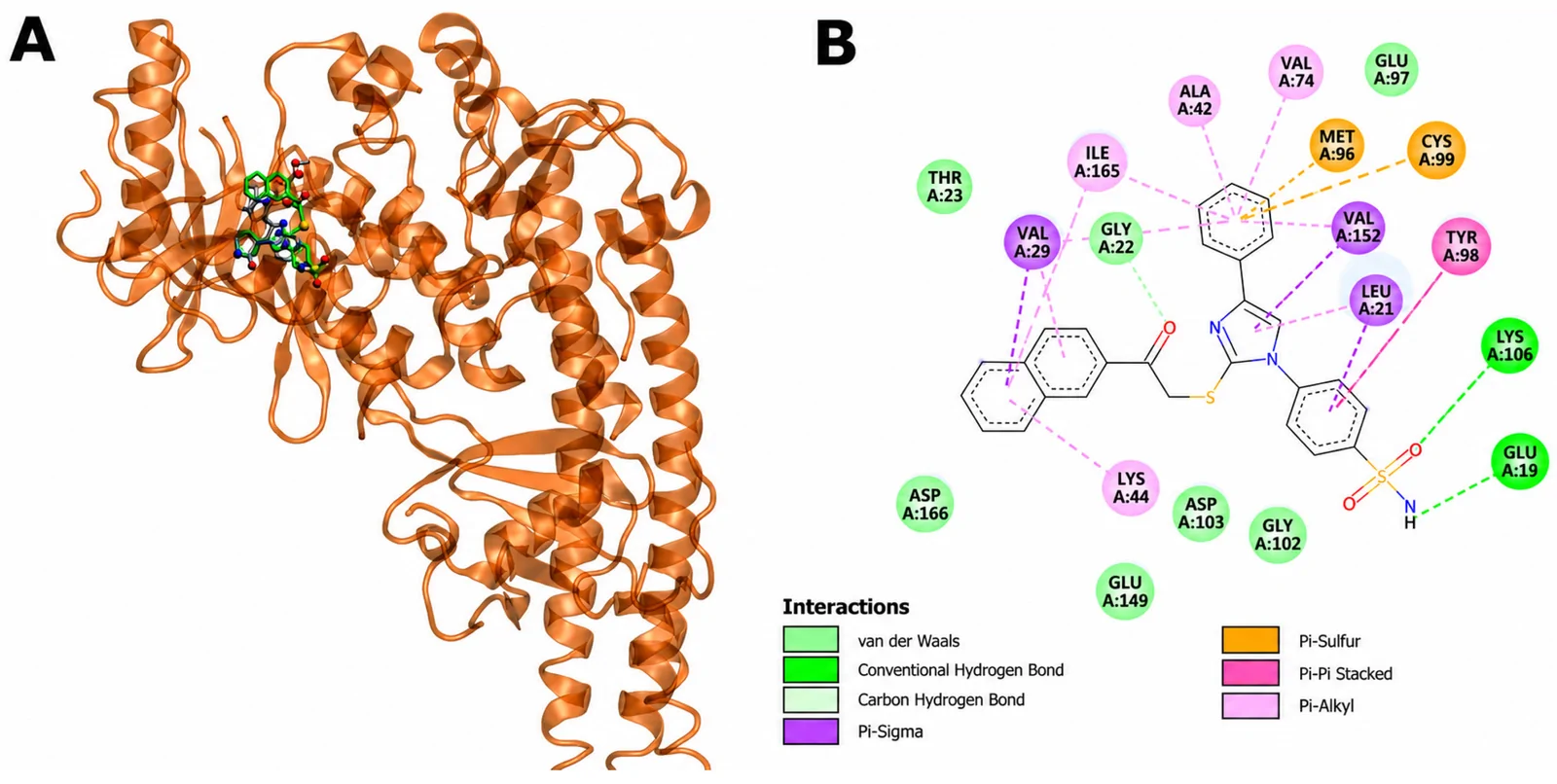
Predicted interaction of compound **1239** with IKK. (**A**) Three-dimensional representation of the predicted binding pose of compound **1239** within the IKK binding site, shown together with the crystallographic ligand. Visualization of the potential binding site of compound **1239** (in green) and the ligand (in silver). (**B**) Two-dimensional interaction map showing the predicted contacts between compound **1239** and residues within the IKK binding site. Hydrogen-bond, van der Waals, and hydrophobic interactions are indicated.

Considering the pronounced activity of compound **1239** in the macrophage assays, its predicted interactions with IKK and p38 MAPK were examined in greater detail. Compound **1239** was positioned within the IKK ligand-binding region and shared several interacting residues with the crystallographic ligand, including Leu21, Gly22, Thr23, Val29, Ala42, Lys44, Val74, Met96, Glu97, Tyr98, Cys99, Gly102, Asp103, Glu149, Val152, Ile165, and Asp166. The docking model further predicted hydrogen-bond interactions between **1239** and Glu19 and Lys106 (Figure 8A,B) (Table S2).

**Figure 9:**
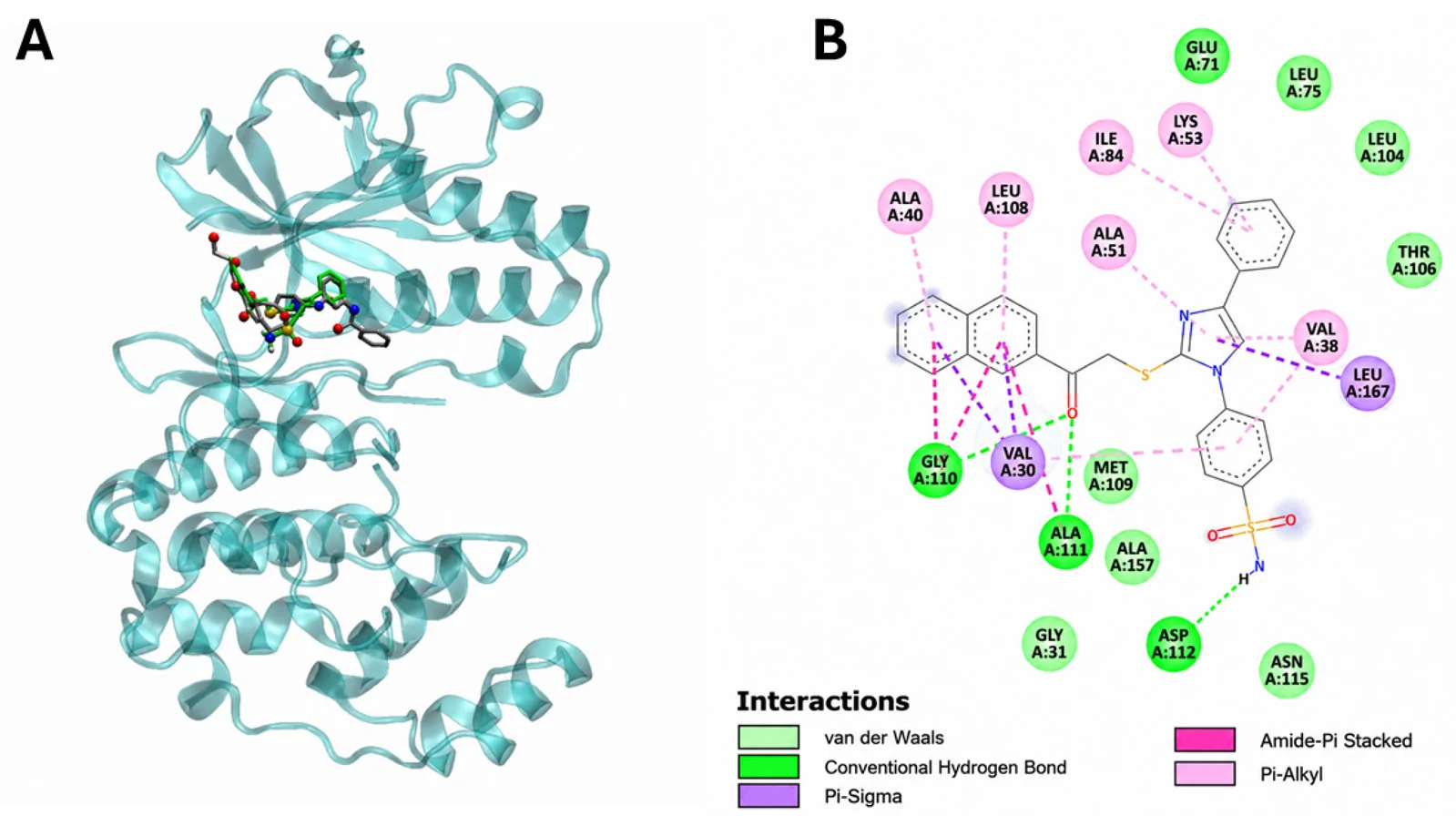
Predicted interaction of compound **1239** with p38 MAPK. (**A**) Three-dimensional representation of the predicted binding pose of compound **1239** within the p38 MAPK binding site, shown together with the crystallographic ligand. Visualization of the potential binding site of compound **1239** (in green) and the ligand (in silver). (**B**) Two-dimensional interaction map showing the predicted contacts between compound **1239** and residues within the p38 MAPK binding site. Hydrogen-bond, van der Waals, and hydrophobic interactions are indicated.

Docking of compound **1239** to p38 MAPK similarly positioned the molecule within the ligand-binding region. Several residues involved in the predicted interaction with **1239** were also observed for the crystallographic ligand, including Val30, Val38, Ala40, Ala51, Lys53, Glu71, Leu75, Ile84, Leu104, Thr106, Leu108, Met109, Gly110, Ala111, Asp112, Asn115, and Leu167. The model predicted hydrogen-bond interactions involving Gly110, Ala111, and Asp112 (Figure 9; Table S2).

Together, the docking analysis suggests that compound **1239** can adopt favorable predicted binding poses within IKK and p38 MAPK binding sites. Given the established involvement of these kinases in LPS-induced inflammatory signaling, these interactions provide plausible candidates for further investigation of the mechanism underlying the observed effects on NF-κB activation and inflammatory mediator production. The docking results, however, represent predicted molecular interactions and do not establish direct target engagement.

## 3. Discussion

Inflammation is an essential component of host defense and tissue repair; however, excessive or persistent inflammatory signaling contributes to the pathogenesis of numerous chronic and acute disorders [27]. NF-κB occupies a central position in this response by coordinating the expression of cytokines, chemokines, inducible enzymes, and other mediators of innate immunity. Persistent or dysregulated NF-κB activity has consequently been implicated in inflammatory, autoimmune, malignant, and other pathological conditions [27,28]. This has generated considerable interest in small molecules capable of modulating NF-κB-dependent responses. At the same time, the broad physiological functions of NF-κB make complete pathway blockade undesirable and emphasize the need to identify chemically diverse modulators with defined activity profiles.

In the present study, screening of an in-house library of aromatic and heterocyclic drug-like molecules identified eight compounds that strongly reduced LPS-induced NF-κB reporter activity. Cytotoxicity counter-screening subsequently narrowed this group to five 4-phenylimidazole-based benzenesulfonamide derivatives, **1232**, **1239**, **1241**, **1242**, and **1245**. Importantly, their activity was not restricted to the initial reporter assay. The selected compounds reduced LPS-induced nitrite production in RAW 264.7 macrophages and altered NF-κB nuclear localization, while individual derivatives produced distinct effects on inflammatory cytokine secretion. Together, these findings support the identification of this chemical series as a starting point for further investigation of small-molecule modulators of inflammatory signaling.

Differences among the five derivatives also suggest that the sulfur-linked substituent influences biological activity. Compound **1239**, bearing a naphthyl-substituted thioacetyl group, showed the strongest overall activity, particularly against nitrite, IL-6, and KC/CXCL1 production. Compound **1232**, containing the smaller ethylthio-substituent, showed a distinct profile with stronger inhibition of TNF-α. The 4-trifluoromethylphenyl derivative **1241** strongly reduced nitrite production but had a more limited effect on cytokine secretion. These differences indicate preliminary structure-dependent modulation of the inflammatory response, although the current compound set is too small to establish a formal structure–activity relationship. Heterocyclic systems containing imidazole or related azoles have previously provided useful chemical frameworks for anti-inflammatory compound development [16]. For example, imidazol-5-yl pyridine derivatives developed as p38α/MAPK14 inhibitors were reported to suppress NO and pro-inflammatory cytokine production in LPS-stimulated RAW 264.7 macrophages. Similarly, introduction of an imidazole-containing substituent into emodin derivatives yielded compounds capable of reducing NO, iNOS, COX-2, IL-6, IL-1β, and TNF-α and interfering with NF-κB signaling in LPS-stimulated RAW 264.7 cells [17].

The non-uniform effects on IL-6, KC/CXCL1, and TNF-α are consistent with the complexity of LPS-induced inflammatory signaling. Although NF-κB contributes to the transcriptional regulation of these mediators, their expression is also influenced by MAPK-dependent pathways and other transcriptional and post-transcriptional regulatory mechanisms. Differences in the relative contribution and kinetics of these pathways may therefore result in compound- and mediator-specific responses. Accordingly, modulation of NF-κB-associated signaling would not necessarily be expected to suppress IL-6, KC/CXCL1, and TNF-α to the same extent. The distinct cytokine profiles observed among the compounds may also suggest that individual derivatives affect different components of the broader LPS-induced signaling network. KC/CXCL1 [29,30,31]. Therefore, inhibition of NF-κB signaling would not necessarily be expected to suppress all cytokines to the same extent.

The molecular docking analysis provided additional insight into signaling pathways that may contribute to the observed anti-inflammatory activity. Among the prioritized derivatives, compound **1239** showed favorable predicted interactions with both IKK and p38 MAPK, with docking scores of −10.3 kcal/mol for each protein. Compound **1239** occupied binding regions overlapping those of the corresponding crystallographic ligands and formed predicted hydrogen-bond interactions within both kinase binding sites. These findings are consistent with the experimental phenotype, as modulation of IKK could account for the observed reduction in NF-κB activation and nuclear localization, while interaction with p38 MAPK may contribute to the effects on downstream inflammatory mediators [26,29,32]. The potential involvement of more than one signaling pathway may also explain the differential effects observed on nitrite and individual cytokines [29]. However, molecular docking provides only a prediction of potential protein–ligand interactions and does not demonstrate direct target engagement [33]. Biochemical assessment of IKK and p38 MAPK activity, together with analysis of pathway phosphorylation, will therefore be required to determine whether these kinases contribute directly to the activity of compound **1239**.

Several limitations should be considered. The compounds were initially evaluated at 100 μM, and some residual cytotoxicity was observed, making concentration-response studies necessary to determine potency and selectivity. The molecular target of the compounds also remains unknown; the present data demonstrate interference with LPS-induced NF-κB signaling but do not establish direct NF-κB inhibition. Additional analysis of IκBα degradation, IKK and p65 phosphorylation, and MAPK signaling would help define the affected pathway [32]. The present study was not designed to evaluate cyclooxygenase selectivity, and the inclusion of COX-2 in the docking panel should not be interpreted as evidence that these compounds act as COX inhibitors. Dedicated COX-1/COX-2 biochemical assays and target-engagement studies would be required to address this question. Finally, confirmation in primary human macrophages and expansion of the analogue series would be required to establish translational relevance and more robust structure–activity relationships.

Overall, these findings identify a 4-phenylimidazole-based benzenesulfonamide series with reproducible anti-inflammatory activity across several experimental readouts. The observed substituent-dependent differences provide a basis for further chemical optimization and mechanistic studies aimed at defining the molecular targets and improving the activity profile of this compound class.

## 4. Materials and Methods

### 4.1. Cell Lines and Culture Conditions

THP-1 Dual™ NF-κB reporter cells (InvivoGen, San Diego, CA, USA) were propagated in RPMI-1640 (Gibco, Thermo Fisher Scientific, Waltham, MA, USA) containing 10% heat-inactivated fetal bovine serum (FBS; Sigma-Aldrich, St. Louis, MO, USA), 1% penicillin-streptomycin (Gibco, Thermo Fisher Scientific), and 100 µg/mL Normocin™ (InvivoGen). Cells were maintained in suspension under standard culture conditions, with fresh medium provided twice per week.

RAW 264.7 murine macrophages (ATCC^®^ TIB-71™, American Type Culture Collection, Manassas, VA, USA) were maintained in RPMI-1640 supplemented with 10% heat-inactivated FBS and 1% penicillin-streptomycin. Cells were grown as adherent monolayers and subcultured twice weekly.

### 4.2. Small-Molecule Screening Library Preparation

The in-house small-molecule library was kindly provided by Dr. Vidmantas Petraitis (Center for Discovery and Innovation, New Jersey, USA) and comprised a collection of structurally diverse aromatic and heterocyclic drug-like compounds [18,34,35]. Individual compounds were dissolved in dimethyl sulfoxide (DMSO) (Sigma-Aldrich, St. Louis, MO, USA) to prepare concentrated stock solutions and stored until use. For screening experiments, compounds were diluted in cell culture medium to the required working concentration immediately before treatment. The final DMSO concentration was kept constant across experimental conditions, with an equivalent concentration of DMSO used as the vehicle control.

### 4.3. Small-Molecule Library Screening in THP-1 Dual Cells

For screening experiments, THP-1 Dual cells were collected by centrifugation (1000× *g*, 5 min), washed twice with complete RPMI-1640 medium without Normocin™, and resuspended in antibiotic-free complete medium. Cells were seeded in flat-bottom 96-well tissue culture plates (Corning, Corning, NY, USA) at 2 × 10^4^ cells per well in a final volume of 100 µL and incubated overnight at 37 °C in a 5% CO_2_ atmosphere.

Library compounds were diluted from DMSO stocks in antibiotic-free complete medium and added to the cells at a final concentration of 100 µM. A concentration of 100 μM was selected for the primary single-concentration screen to facilitate detection of compounds with measurable NF-κB-modulatory activity across the chemically diverse library; potential cytotoxic effects at this concentration were subsequently evaluated by LDH release. An equivalent concentration of DMSO was used as the vehicle control. Cells were pre-incubated with the compounds for 3 h before stimulation with lipopolysaccharide (LPS) from *Escherichia coli* O111:B4 (Sigma-Aldrich, St. Louis, MO, USA) at a final concentration of 100 ng/mL. Following LPS addition, cells were incubated for a further 24 h under standard culture conditions.

Plates were subsequently centrifuged at 1000× *g* for 5 min, and culture supernatants were transferred to fresh 96-well plates. NF-κB reporter activity was determined using QUANTI-Blue™ reagent (InvivoGen) according to the manufacturer’s protocol. Reporter activity was measured spectrophotometrically and normalized to the LPS-stimulated DMSO control, which was defined as 100% NF-κB activation. Compounds reducing NF-κB activity to below 25% of the LPS control were selected for further evaluation.

### 4.4. LDH Assay for Cytotoxicity Determination

Compound-associated cytotoxicity was evaluated by measuring lactate dehydrogenase (LDH) [36] release using the CyQUANT™ LDH Cytotoxicity Assay (Thermo Fisher Scientific, Waltham, MA, USA). Cells were treated with the selected compounds at 100 µM under the same conditions used for the NF-κB screening assay and macrophage stimulation. Following incubation, culture supernatants were collected, and LDH activity was determined according to the manufacturer’s instructions. Untreated cells served as the negative control, while the kit-provided lysis condition was used as the maximum LDH release control. Cytotoxicity was expressed relative to the maximum LDH release control.

### 4.5. Immunofluorescence

RAW 264.7 macrophages were seeded onto sterile glass coverslips and allowed to adhere overnight. Cells were pretreated with the indicated compounds (100 µM) for 1 h and subsequently stimulated with LPS (100 ng/mL) for 3 h. Following stimulation, cells were washed with PBS, fixed with paraformaldehyde (PFA), and permeabilized with 1% Triton X-100 in PBS for 10 min at room temperature. Non-specific binding was blocked with 5% bovine serum albumin (BSA) in PBS.

Cells were incubated for 1 h with NF-κB p65 (D14E12) rabbit monoclonal antibody (Cell Signaling Technology, Danvers, MA, USA; Cat. No. 8242), followed by washing and incubation with Alexa Fluor 488-conjugated donkey anti-rabbit IgG (Invitrogen, Thermo Fisher Scientific; Cat. No. A-21206). Nuclei were counterstained with Hoechst 33342 (Invitrogen, Thermo Fisher Scientific; Cat. No. H3570). Coverslips were mounted onto glass slides using antifade mounting medium and imaged by fluorescence microscopy. Images were acquired using identical acquisition settings across experimental conditions.

### 4.6. Cytokine Quantification by Enzyme-Linked Immunosorbent Assay

RAW 264.7 macrophages were seeded into 6-well tissue culture plates and allowed to adhere overnight. Cells were pretreated with compounds **1232**, **1239**, **1241**, **1242**, and **1245** at a final concentration of 100 µM for 1 h, followed by stimulation with LPS (100 ng/mL) for 24 h. When needed, THP-1-derived macrophages were also used. DMSO-treated cells were used as vehicle controls, and unstimulated and LPS-stimulated controls were included in each experiment. Following incubation, conditioned media were collected, clarified by centrifugation to remove cells and cellular debris, and stored at −80 °C until analysis.

Cytokine concentrations were determined using commercially available mouse immunoassays according to the manufacturers’ instructions. IL-6 was measured using the Mouse IL-6 Uncoated ELISA kit (Invitrogen, Thermo Fisher Scientific, Waltham, MA, USA; Cat. No. 88-7064-88), KC/CXCL1 using the KC/CXCL1 ELISA kit (Invitrogen, Thermo Fisher Scientific; Cat. No. KAC1301), mouse KC/CXCL1 using the Mouse KC/CXCL1 ELISA Kit (Cat. EMCXCL1), and TNF-α using the Mouse TNF-α Uncoated ELISA kit (Invitrogen, Thermo Fisher Scientific; Cat. No. 88-7324-88). Cytokine concentrations were calculated from the corresponding standard curves and expressed as pg/mL.

### 4.7. THP-1 Cell Differentiation to Macrophage-Like Phenotype

For differentiation to a macrophage phenotype, THP-1 Dual cells were treated with 200 nM calcitriol (1,25-dihydroxyvitamin D3; MedChemExpress, Monmouth Junction, NJ, USA; HY-10002) for 48 h at 37 °C in a humidified incubator with 5% CO_2_. Following differentiation, cells were washed, trypsinized, and seeded onto either 6-well plates for cytokine ELISA experiments or 96-well plates for the NF-κB reporter assay.

### 4.8. In Silico Modeling

#### 4.8.1. Receptor Preparation

The crystal structures of 11 selected proteins were retrieved from the Protein Data Bank. These proteins, including cyclooxygenase-2 (COX-2), receptor tyrosine kinases, MAPK/ERK kinase, NF-κB-inducing kinase (NIK), and transcription factors, play key roles in the regulation of inflammatory processes.

#### 4.8.2. Ligand Preparation

The structures of the compounds **1232**, **1239**, **1241**, **1242,** and **1245** were converted from SMILES to MOL2 using OpenBabel (version 3.1.1) and geometrically optimized using Avogadro (version 2.0.0) [37]. These structures were visually checked to correct some structural errors. The 3D structures of the ligands were extracted from the crystal structures 4KIK and 3UVQ.

#### 4.8.3. Docking of Ligand–Protein Interaction

The compounds were docked into proteins to identify their potential binding sites. Both the ligand and protein were prepared using AutoDock Tools version 1.5.7. Docking calculations were performed using AutoDock Vina (version 1.2.0) [38,39]. Gasteiger and Kollman partial charges were assigned to the atoms of the ligand and protein, respectively. The AutoTors option was used to define the rotatable bonds in the ligand. We selected a grid size large enough to cover each receptor. Finally, graphical analysis was performed using VMD [40] and BIOVIA Discovery Studio.

### 4.9. Statistical Analysis

Statistical analyses were performed using GraphPad Prism version 11 (GraphPad Software, Boston, MA, USA). Data are presented as mean ± standard deviation (SD) from three independent replicates unless otherwise indicated. Comparisons between multiple experimental groups were performed using one-way analysis of variance (ANOVA) followed by Dunnett’s multiple-comparisons test, with each treatment group compared with the corresponding LPS-stimulated control. A *p*-value < 0.05 was considered statistically significant. Statistical significance is indicated as * *p* < 0.05, ** *p* < 0.01, *** *p* < 0.001, and **** *p* < 0.0001.

## 5. Conclusions

This study identified a series of 4-phenylimidazole-based benzenesulfonamide derivatives with anti-inflammatory activity in LPS-stimulated cellular models. Five compounds (**1232**, **1239**, **1241**, **1242**, and **1245**) suppressed NF-κB reporter activation while exhibiting lower cytotoxicity than the other primary screening hits. Their activity was further supported by reduced nitrite production, altered NF-κB nuclear localization, and compound-dependent modulation of inflammatory cytokine production in RAW 264.7 macrophages. Among the prioritized derivatives, compound **1239** showed the broadest overall anti-inflammatory profile, whereas compound **1232** displayed a distinct effect on TNF-α production, suggesting that substitution at the sulfur-linked position influences biological activity.

Although the present findings support these compounds as promising modulators of inflammatory signaling, their direct molecular targets remain to be established. The docking analysis provides only hypothesis-generating evidence for possible interactions with inflammation-associated signaling proteins and should not be interpreted as confirmation of target engagement. Future studies should therefore focus on concentration-response relationships, biochemical and target-engagement assays, pathway-level validation of IKK/NF-κB and MAPK signaling, evaluation in primary human immune cells, and expansion of the analogue series to define more robust structure–activity relationships. These studies will be important for determining the potency, selectivity, and translational potential of this compound class.

## Figures and Tables

**Table 1:** Predicted binding free energy values (∆G_bin_ kcal/mol) of identified compounds.

Compounds	Target Proteins									
	COX-2	TLR4	MyD88	IKK	p38	NF-κβ	ERK2	MEK1	TRKA	TPK
**1232**	−9.6	−6.3	−6.8	−8.7	−8.2	−6.5	−8.0	−8.4	−8.8	−8.9
**1239**	−8.3	−7.3	−8.1	−10.3	−10.3	−7.8	−9.8	−11.3	−10.9	−9.5
**1241**	−7.4	−6.9	−7.4	−9.6	−9.7	−7.7	−9.9	−11.0	−11.0	−9.6
**1242**	−11.0	−6.6	−7.9	−9.6	−9.1	−8.7	−9.7	−10.5	−10.4	−9.6
**1245**	−10.1	−6.3	−6.4	−8.1	−8.2	−6.8	−8.2	−8.7	−8.6	−7.5
P avge.	−9.30	−6.71	−7.31	−9.29	−9.08	−7.51	−9.12	−9.98	−9.93	−9.01

Proteins with their respective (PDB) entries: COX-2: Cyclooxygenase 2 (3LN1); TLR: Toll-like Receptor (3FXI); MyD88: Myeloid Differentiation Primary Response 88 (4DOM); IKK: IκB Kinase (4KIK); p38: p38 MAP Kinase (3UVQ); NF-κβ: Factor Nuclear kappa β (1A3Q), ERK2: Extracellular Signal-regulated Kinase 2 (2OJG); MEK1: Mitogen-activated Protein Kinase Kinase 1 (4AN3); TRKA: Tyrosine Kinase Receptor (6PL2), TPK: Tyrosine-protein Kinase (4EHZ). P avge. (protein average): mean of the ∆G_bin_ values for the interactions of each protein with all the hybrids; the two proteins with the highest global compound affinity are highlighted in red colour.

## Data Availability

All data generated or analyzed during this study are provided within the manuscript and its Supplementary Materials.

## Supplementary Materials

The following supporting information can be downloaded at https://www.mdpi.com/article/10.3390/molecules31193472/s1.
