# Supplementary material for: Identification of Imidazole–Benzenesulfonamide Derivatives with Anti-Inflammatory Activity Through Modulation of NF-κB Signaling
Source: Molecules. 2026 Sep 29;31(19):3472. doi: 10.3390/molecules31193472 (PMC13666921; doi:10.3390/molecules31193472)
Supplement: Supplementary file 1 [file molecules-31-03472-s001.zip › molecules-4540518-supplementary.pdf]

Identification of Imidazole–Benzenesulfonamide Derivatives with Anti-Inflammatory Activity through Modulation of NF-κB Signaling

SUPLEMENTARY INFORMATION

Povilas Kavaliauskas [1,2,3,4], Valdas Vainauskas [2], Vidmantas Petraitis [3,5], Ruta Petraitiene [3,5], Waldo Acevedo [6,7], Ramunė Grigalevičiūtė [4,8], Birutė Grybaitė [2], Božena Golcienė [2], Rolandas Stankevičius [8], Julius Rakickas [9] and Vytautas Mickevičius [2,*]

[1] Institute for Genome Sciences, School of Medicine, University of Maryland, 655 W. Baltimore Street, Baltimore, MD 21201, USA; povilas.kavaliauskas@som.umaryland.edu

[2] Department of Organic Chemistry, Kaunas University of Technology, Radvilėnų Rd. 19, LT-50254 Kaunas, Lithuania

[3] Institute of Infectious Diseases and Pathogenic Microbiology, Birštono Str. 38A, LT-59116 Prienai, Lithuania

[4] Biological Research Center, Lithuanian University of Health Sciences, Tilzes Str. 18/7, LT-47181 Kaunas, Lithuania

[5] Center for Discovery and Innovation, Hackensack Meridian Health, Nutley, NJ 07110, USA

[6] Institute of Chemistry, Faculty of Sciences, Pontificia Universidad Católica de Valparaíso, Valparaíso 2373223, Chile; waldo.acevedo@pucv.cl

[7] Center for Interdisciplinary Research in Biomedicine, Biotechnology and Well-Being (CID3B), Pontificia Universidad Católica de Valparaíso, Valparaíso 2340025, Chile

[8] Department of Animal Nutrition, Lithuanian University of Health Sciences, Tilzes Str. 18, LT-47181 Kaunas, Lithuania

[9] Public Institution Šilutė Primary Health Care Center, Rusnes Str. 1, LT-99185 Šilutė, Lithuania

* Correspondence: vytautas.mickevicius@ktu.lt

Table S1. The representation of sulfonamide-imidazole hybrid derivatives previously published by Vainauskas *et al.,* [20] sharing structural similarity and were incorporated to the screening library. The compound number represents compounds published in original paper [14] while numbers in brackets represents code in screening plates of hit compounds.

No.	Compound	No.	Compound
**2**	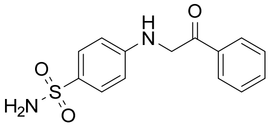	**11d**	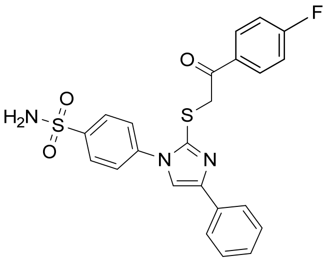
**3**	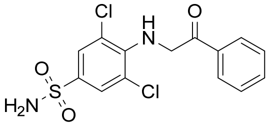	**11e (1239)**	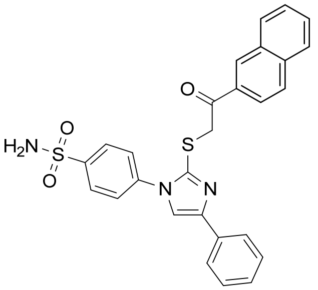
**4a**	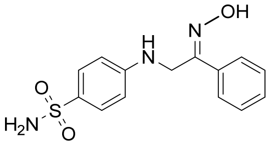	**11f**	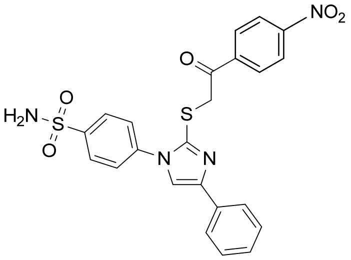
**4b**	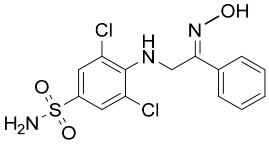	**11g (1241)**	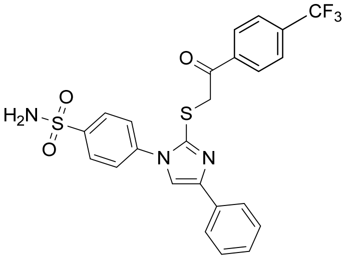

**5**	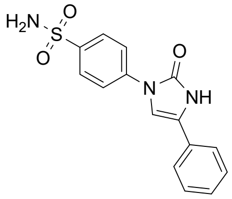	**11h (1242)**	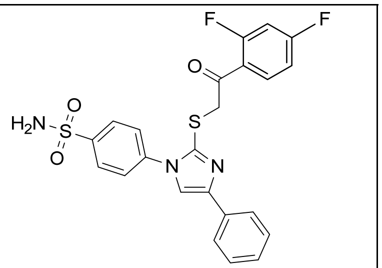
**6**	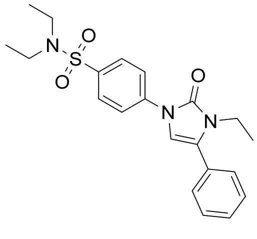	**11i**	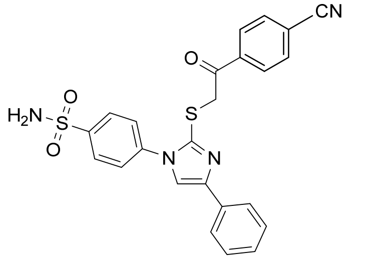
**7**	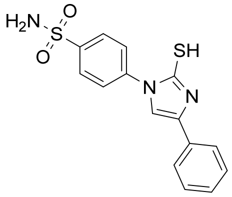	**11j**	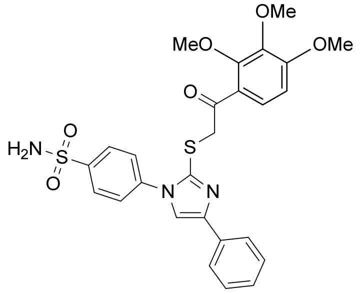
**8 (1235)**	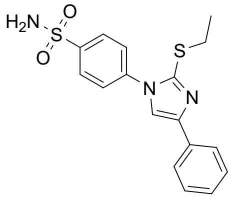	**12 (1245)**	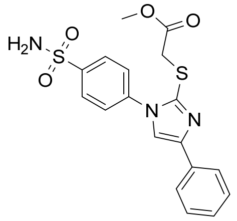
**9**	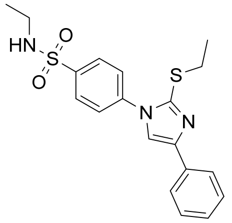	**13**	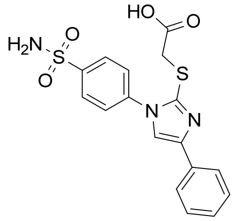
**10**	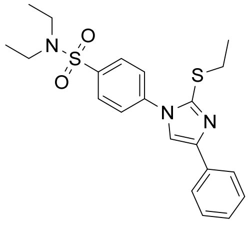	**14a (1247)**	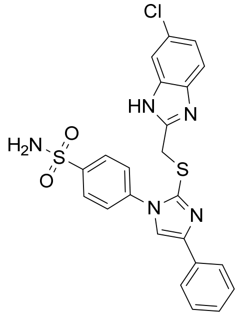

**11a**	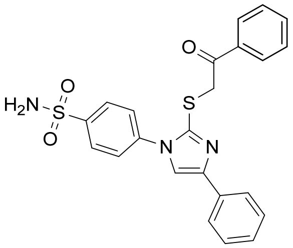	**14b (1248)**	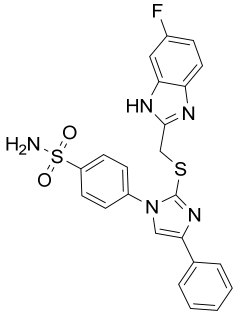
**11b**	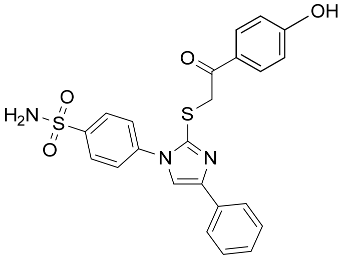	**15 (1249)**	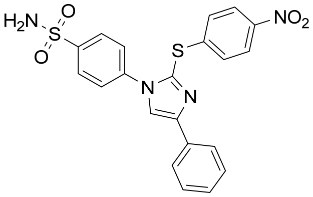
**11c**	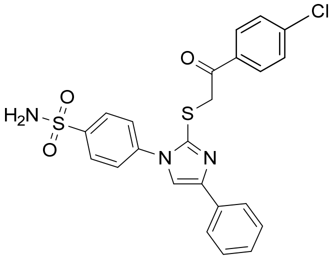		

Table S2. Binding site contacts of compound **1239**, and ligands into IKK and p38.

**Compound**	**ΔG_{bin} (kcal/mol)**	**H bonds and hydrophobic contacts in the binding site**
		**IKK**
**1239**	-10.3	**Glu19**, Leu21, Gly22, Thr23, Val29, Ala42, Lys44, Val74, Met96, Glu97, Tyr98, Cys99, Gly102, Asp103, **Lys106**, Glu149, Val152, Ile165, Asp166

**Ligand 1** [a]	-12.4	Leu21, Gly22, **Thr23**, Val29, Ala42, Lys44, Val74, Met96, **Glu97**, Tyr98, **Cys99**, Gly102, Asp103, Glu149, Val152, Ile165, Asp166
		**p38 MAP Kinase**
**1239**	-10.3	Val30, Gly31, Val38, Ala40, Ala51, Lys53, **Glu71**, Leu75, Ile84, Leu104, Thr106, Leu108, Met109, **Gly110**, **Ala111**, **Asp112**, Asn115, Ala157, Leu167
**Ligand 2** [a]	-12.3	Val30, Val38, Ala40, **Arg49**, Ala51, Lys53, Glu71, Leu74, Leu75, Ile84, Leu104, Thr106, Leu108, **Met109**, Gly110, Ala111, Asp112, Asn115, Leu167, **Asp168**, Phe169

[a] Ligands 1 and 2 correspond to K-252A and N-{5-[(7-{[(2R)-2,3-dihydroxypropyl]oxy}-5-oxo-10,11-dihydro-5H-dibenzo[a,d][7]annulen-2-yl)amino]-2-fluorophenyl}benzamide, respectively. The three-dimensional structure of ligands 1 and 2 was extracted from the Protein Data Bank using PDB IDs 4KIK and 3UVQ, respectively. [b] Drug compound that act as inhibitors of biological targets. Words colored in red correspond to amino acids shared by **1239** and ligands. Bolded names correspond to amino acids involved in H bonds.

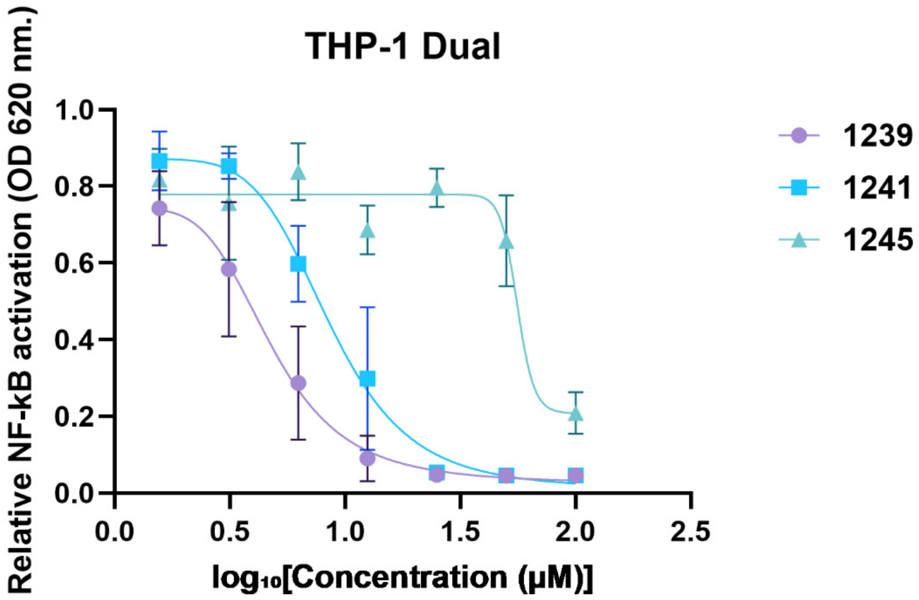

Figure S1. Concentration–response curves for inhibition of NF-κB activation by compounds 1239, 1241, and 1245 in THP-1 Dual reporter cells. The cells were treated with compounds for 1 hour and stimulated overnight with LPS. After stimulation, NFkB activation was measured as described in materials and methods. Relative NF-κB activity was measured at 620 nm following treatment with increasing compound concentrations. Data are presented as mean ± SD from tree (n=3) technical replicates.
